# Systematic Surveillance Detects Multiple Silent Introductions and Household Transmission of Methicillin-Resistant *Staphylococcus aureus* USA300 in the East of England

**DOI:** 10.1093/infdis/jiw166

**Published:** 2016-04-27

**Authors:** Michelle S. Toleman, Sandra Reuter, Francesc Coll, Ewan M. Harrison, Beth Blane, Nicholas M. Brown, M. Estée Török, Julian Parkhill, Sharon J. Peacock

**Affiliations:** 1 University of Cambridge; 2 Wellcome Trust Sanger Institute; 3 Cambridge University Hospitals NHS Foundation Trust; 4 Clinical Microbiology and Public Health Laboratory, Public Health England,Cambridge; 5 London School of Hygiene and Tropical Medicine, United Kingdom

**Keywords:** molecular epidemiology, whole-genome sequencing, *Staphylococcus aureus*, MRSA, USA300

## Abstract

***Background.*** The spread of USA300 methicillin-resistant *Staphylococcus aureus* (MRSA) across the United States resulted in an epidemic of infections. In Europe, only sporadic cases or small clusters of USA300 infections are described, and its prevalence in England is unknown. We conducted prospective surveillance for USA300 in the east of England.

***Methods.*** We undertook a 12-month prospective observational cohort study of all individuals with MRSA isolated from community and hospital samples submitted to a microbiology laboratory. At least 1 MRSA isolate from each individual underwent whole-genome sequencing. USA300 was identified on the basis of sequence analysis, and phylogenetic comparisons were made between these and USA300 genomes from the United States.

***Results.*** Between April 2012 and April 2013, we sequenced 2283 MRSA isolates (detected during carriage screening and in clinical samples) from 1465 individuals. USA300 was isolated from 24 cases (1.6%). Ten cases (42%) had skin and soft tissue infection, and 2 cases had invasive disease. Phylogenetic analyses identified multiple introductions and household transmission of USA300.

***Conclusions.*** Use of a diagnostic laboratory as a sentinel for surveillance has identified repeated introductions of USA300 in eastern England in 2012–2013, with evidence for limited transmission. Our results show how systematic surveillance could provide an early warning of strain emergence and dissemination.

Identification of the community-associated methicillin-resistant *Staphylococcus aureus* (CA-MRSA) pulsotype USA300 in the United States in 1999 was followed by widespread dissemination and an epidemic of MRSA infection in otherwise healthy people [1, 2]. By 2004, this lineage was responsible for up to 97% of skin and soft-tissue infections (SSTIs) among individuals presenting to US emergency departments [3]. USA300 also causes invasive disease, such as pneumonia and osteomyelitis, and has become endemic in US hospitals, where it causes hospital-associated infections, including bacteremia [4, 5]. USA300 is readily transmitted within households, which act as long-term reservoirs associated with repeated episodes of infection and onward transmission [6, 7]. Antimicrobial resistance to macrolides and fluoroquinolones is common [3]. An epidemic of CA-MRSA infection caused by a clone that is closely related to USA300 but arose independently (USA300 Latin American variant [USA300-LV]) has also been identified in South America [8].

International travel is an important contributor to the intercontinental spread of infectious diseases [9], and the spread of USA300 and USA300-LV have been documented globally [1, 10]. In the United Kingdom, the vast majority of MRSA infections continue to be caused by the dominant hospital-associated lineage EMRSA-15 (ST22) [11]. USA300 is considered of low prevalence in continental Europe, presenting primarily as sporadic cases and in discrete, small outbreaks [12–18]. Similarly, sporadic cases and a single hospital outbreak caused by USA300 have been described in the United Kingdom [19–22], but the prevalence of USA300 carriage and infection is unknown. In the absence of systematic molecular surveillance of high-risk lineages, we hypothesized that USA300 would only become apparent in England if a high prevalence of SSTIs among patients presenting to emergency departments triggered concern. Here, we report the findings of systematic surveillance for USA300 in a major diagnostic microbiology laboratory serving part of eastern England.

## METHODS

### Study Design

A prospective study was conducted over 12 months between April 2012 and April 2013 at the Public Health England Clinical Microbiology and Public Health Laboratory, Cambridge University Hospitals NHS Foundation Trust in Cambridge, United Kingdom. This laboratory processes samples submitted by 4 Cambridgeshire hospitals (Addenbrooke's Hospital, the Rosie Hospital, Papworth Hospital, and Hinchingbrooke Hospital) and providers of community healthcare in the same geographic region. We identified all cases with MRSA isolated at least once from screening swabs and/or clinical specimens and collected demographic and clinical data on MRSA-positive cases, using electronic and paper medical records. Throughout the study period, universal MRSA screening was performed at the 4 hospitals, in accordance with national policy (multisite MRSA screening of all patients on hospital admission and weekly MRSA screening of patients in critical care units). The study protocol was approved by the National Research Ethics Service (reference 11/EE/0499), the National Information Governance Board Ethics and Confidentiality Committee (reference ECC 8-05(h)/2011), and the Cambridge University Hospitals NHS Foundation Trust Research and Development Department (reference A092428).

### Microbiologic Evaluation, DNA Sequencing, and Analysis

**Figure 1.**
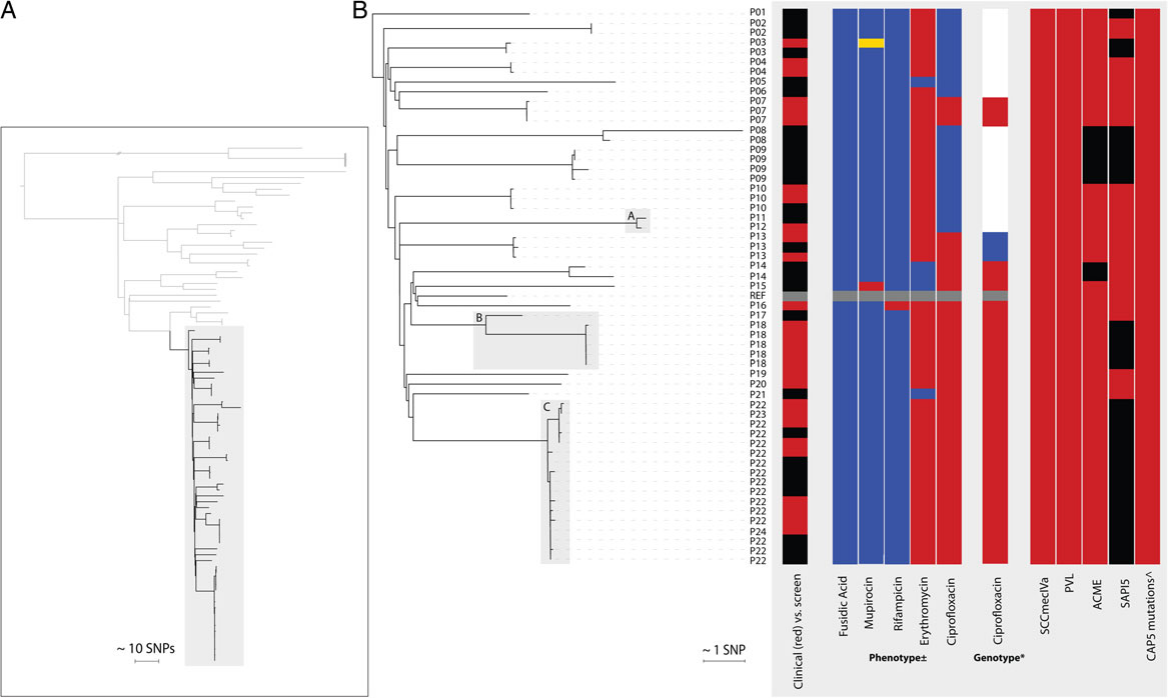
*A*, Phylogenetic, midpoint-rooted tree of study CC8 isolates, with USA300 isolates highlighted. A total of 56 isolates residing in the subclade within the gray box were phylogenetically identified as USA300 isolates. *B*, Detailed USA300 phylogenetic tree rooted on the isolate from P01, with a summary of metadata for each isolate. Person (P) numbers represent the study identifier of each individual from whom the sample was from, with gray boxes indicating pairs or clusters with presumptive epidemiological links. ±Red, resistant; yellow, intermediate; blue, susceptible. *Ciprofloxacin: red, S80Y and S84L; blue, S80F only; white, none identified; gray, not done. Staphylococcal cassette chromosome *mec* (SCC*mec*) IVa, Panton-Valentine leukocidin (PVL), arginine catabolic mobile element (ACME), staphylococcal pathogenicity island 5 (SAPI5): red, present; black, absent. ^Red, 4 mutations associated with the CAP5 locus were identified. Abbreviation: SNP, single-nucleotide polymorphism.

MRSA was isolated from screening samples by plating swabs onto Brilliance MRSA chromogenic medium (Oxoid, Basingstoke, United Kingdom) and from all other samples by plating onto Columbia Blood Agar (Oxoid, Basingstoke, United Kingdom). *S. aureus* was identified using a commercial latex agglutination kit (Pastorex Staph Plus, Bio Rad Laboratories, Hemel Hempstead, United Kingdom). Antimicrobial susceptibility was determined to commonly used antibiotic agents (Figure 1), using the VITEK 2 instrument (bioMerieux, Marcy l′Etoile, France). Inducible clindamycin resistance was detected using the D-test disk diffusion method. Antimicrobial susceptibility results were interpreted using the European Committee on Antimicrobial Susceptibility Testing criteria.

DNA was extracted, libraries prepared, and 150–base pair paired-end sequences determined on an Illumina HiSeq2000 as previously described [23]. Multilocus sequence types (MLSTs) were identified from the sequence data, using an in-house script and the MLST database (available at: http://saureus.mlst.net/), and assigned to clonal complexes (CCs). CC8 isolates were identified and formed the basis for the remaining genetic analyses. CC8 were mapped using SMALT to the *S. aureus* USA300 genome FPR3757 (GenBank accession number CP000255.1). Mobile genetic elements, indels, and regions of high-density single-nucleotide polymorphisms (SNPs) were excluded. SNPs in this core genome were used to create maximum likelihood phylogenies, using RAxML with 100 bootstraps [24]. Genome sequence data for 348 MRSA isolates reported previously by Uhlemann et al were sourced from the European Nucleotide Archive. Trees were visualized using FigTree (available at: http://tree.bio.ed.ac.uk/software/figtree/) and iTOL [25]. Sequence data for the USA300 isolates from eastern England have been submitted to the European Nucleotide Archive under the accession numbers listed in Supplementary Table S1. In silico polymerase chain reaction analysis was used to determine the staphylococcal cassette chromosome *mec* (SCC*mec*) subtype [26]. To detect the presence of Panton-Valentine leukocidin (PVL) genes (*lukF-PV* and *lukS-PV*), the arginine catabolic mobile element (ACME), and the staphylococcal pathogenicity island 5 (SAPI5), corresponding sequences were extracted from the mapping alignment. Mutations in the *cap5* locus were retrieved from the assemblies by comparison to the *cap5* locus of the CP5+ reference strain Newman and USA300 reference strains TCH1516 and FPR3757, as described previously [27]. The presence or absence of acquired genes and SNPs conferring resistance against quinolones (*grlA*, *grlB*, *gyrA*, and *gyrB*) were determined as described previously [23]. For SNPs causing resistance in chromosomal genes, the standard mapping and SNP calling approach was used as described earlier.

## RESULTS

### Description of Cases Positive for USA300

We identified 1465 individuals over a 12-month period with at least 1 sample submitted to the microbiology laboratory at CUH that was culture positive for MRSA, and we sequenced 2283 isolates cultured from multisite screens or clinical samples from these cases. CC8 isolates were identified and further evaluated to identify USA300 (SCC*mec* type IVa, presence of PVL genes, and phylogenetic clustering with USA300 reference genome FPR3757; Figure 1*A*). This identified 56 USA300 isolates from 24 cases (Figure 1*B*), giving a case prevalence among MRSA carriers of 1.6% (24 of 1489). The majority of USA300-positive cases were young (median, 32 years; range, 3–84 years; interquartile range, 25–57 years), and males predominated (16 of 24 [67%]). Over half of first positive samples were submitted from first-opinion services (emergency departments, 8 of 24 [33%]; and general practices, 5 of 24 [21%]), rather than from hospital inpatient stays. Eleven cases had MRSA USA300 identified from carriage screening alone: 11 had MRSA USA300 collected from a SSTI wound specimen, with or without associated carriage, and 1 each had invasive infection resulting in bloodstream infection and osteomyelitis, respectively, with MRSA USA300 identified in blood and bone samples, respectively (Figure 1*B*). Mapping the residential address of the 24 cases demonstrated that 23 cases were scattered across eastern England (Supplementary Figure S1), while the remaining case was a US resident. Dates of collection of the positive samples were distributed throughout the 12-month study period, with a median time of 21 days (range, 0–332 days) between collection of the first and last sequenced isolate (Supplementary Table S1). The finding that MRSA USA300–positive cases were distributed in time and space is suggestive of numerous independent introduction events.

### Genomic Investigation

**Figure 2.**
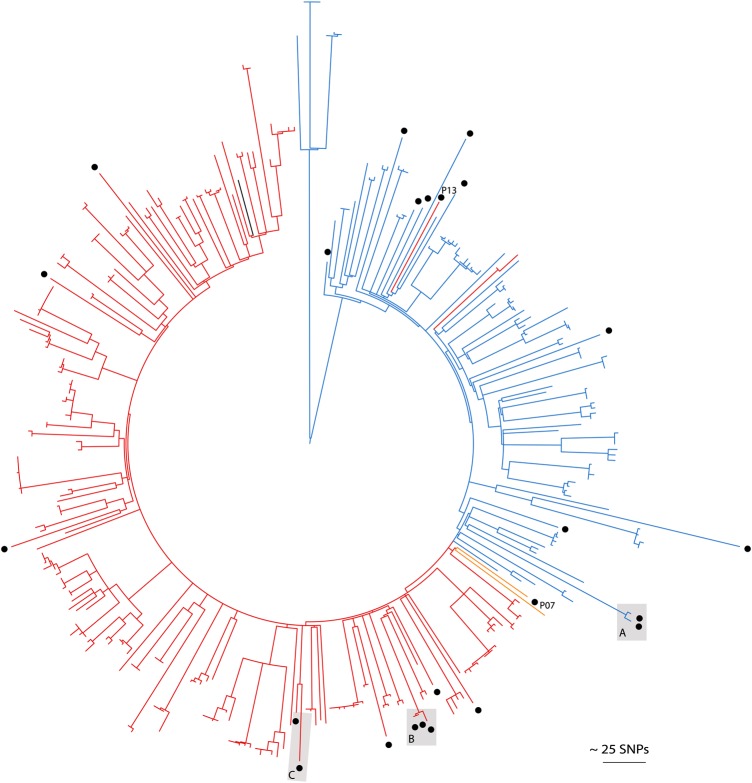
Comparison of the first USA300 isolate from each study case (n = 24, circles) relative to previously published USA300 isolates from the United States (n = 348) [6]. Mid-point rooted maximum likelihood tree based on single-nucleotide polymorphisms (SNPs) in the core genome of methicillin-resistant *Staphylococcus aureus* with branch colors representing fluoroquinolone genotypes. Red branches: S80F/Y and S84A/L; yellow: S80F only; blue branches: nil; black branch: reference genome FPR3757. Letters alongside circles indicate epidemiologically linked pairs or clusters.

The phylogenetic tree revealed 3 groups of closely related isolates containing isolates from >1 individual (termed pairs/clusters A, B, and C; Figure 1). Pair A contained 1 isolate each from 2 cases (persons 11 and 12) that differed by 6 SNPs. Samples were submitted 3 months apart from a general practitioner (ear swab) and a hospital (multisite MRSA screen), and cases shared the same registered address, suggesting household transmission. Pair B contained 6 isolates from 2 cases (persons 17 and 18), which differed by a median of 59 SNPs (range, 57–60 SNPs) but differed from other isolates by a minimum of 81 SNPs. These were isolated from samples collected within 2 weeks of each other and submitted by a general practitioner (multisite screen) and hospital (blood culture), respectively. The 2 cases shared a surname but not address, which, given the larger number of SNPs, potentially suggests spread between family contacts, rather than household transmission. However, this must be interpreted with caution. Cluster C contained 17 isolates from 3 cases (persons 22, 23, and 24) with a median difference of 6 SNPs (range, 0–9 SNPs) between isolates from different individuals. Persons 22 and 24 had the same registered residential address and had 16 MRSA isolates sequenced, the most closely related of which were genetically identical. The single isolate from person 23 was also highly related (the closest genetic distances to isolates from persons 22 and 24 were 1 and 7 SNPs, respectively), but no direct or indirect epidemiological link between persons 22/24 and person 23 could be identified. Overall, we found a maximum genetic distance of 6, 59, and 9 SNPs among epidemiologically linked cases. None of the cases had a recent history of hospitalization in the same ward simultaneously (Supplementary Figure S2). The 56 USA300 genomes from this study were combined with the genomes of 348 MRSA USA300 isolates from New York that have been reported previously [6], to provide genetic context to the United Kingdom isolates. A tree containing all isolates showed that the United Kingdom study genomes were interspersed throughout the tree (Figure 2), suggesting repeated introductions of USA300 and ruling out a single importation and subsequent propagation of a single clone. This picture of multiple introductions is similar to that reported in recent USA300 studies from France and Switzerland [18, 28].

### Variability in the USA300 Genome

We investigated the presence of mobile genetic elements proposed previously to be associated with USA300 fitness and epidemic spread (Figure 1). Enterotoxins K and Q are thought to enhance pathogenesis through T-cell stimulation and are encoded by *sek*2 and *seq*2 within a pathogenicity island, SAPI5 [29, 30]. SAPI5 was present in 25 of 56 isolates. The ACME locus is a genomic island associated with USA300 that is composed of at least 33 putative genes and 2 operons [31]. The *arc* operon encodes genes involved in arginine catabolism, which are important for survival of USA300 in acidic environments [32]. ACME *speG*, which encodes a spermidine acetyltransferase, confers the ability to survive levels of the polyamines spermidine and spermine that are lethal for other strains of *S. aureus* [33]. As described previously [6], ACME was variably present in the USA300 isolates. Eight isolates from 3 cases were missing this gene cassette. The dispersed position of ACME-negative isolates in the phylogeny suggested multiple losses of the preexisting island. ACME-negative isolates did not carry the copper and mercury resistance element, the presence of which would be characteristic of South American strains of USA300-LV [8]. Boyle-Vavra et al recently reported that USA300 failed to produce capsular polysaccharide, which was associated with the presence of 4 conserved mutations associated with the *cap5* locus when compared with strain Newman [27]. We confirmed that these 4 mutations were present in all of our 56 USA300 isolates. These *cap5* mutations, together with SCC*mec* IVa and PVL, therefore form a consistent marker of USA300 in our collection, whereas ACME and SAPI5 are variably present.

### Antimicrobial Resistance

The oral antibiotics used to treat MRSA SSTI in the United Kingdom and United States in single or combination regimens are clindamycin, doxycycline and trimethoprim-sulfamethoxazole, rifampicin, trimethoprim, and fusidic acid [34–36]. All 56 isolates were phenotypically susceptible to trimethoprim and clindamycin (constitutive), and only 1 isolate tested resistant to tetracycline. Of the 51 of 56 erythromycin-resistant isolates, none tested positive for inducible clindamycin resistance. More than half of isolates (36 of 56) were phenotypically resistant to ciprofloxacin and contained known mutations in both *grlA* and *gyrA* (*gyrA* 84L and *grlA* 80Y [n = 33] and *grl*A 80F alone [n = 3]). Previous studies have reported that USA300 isolates from the United States segregated into 2 clades based on fluoroquinolone resistance genotypes (with or without *gyrA* 84L/A and/or *grlA* 80Y/F mutations) [6, 7]. When the study isolates were considered in the context of the US isolates (Figure 2), this was replicated for isolates from 22 cases, the 2 exceptions being isolates from persons 7 and 13. Person 7 carried the 84L/80Y mutations and tested phenotypically resistant but resided within the “susceptible” clade. Person 13 resided at the base of the resistant clade and tested phenotypically resistant but with an 80F mutation within *grl*A.

## DISCUSSION

This study represents, to our knowledge, the first prospective surveillance study for USA300 in the United Kingdom. It provides clear evidence of how systematic whole-genome sequencing might assist in monitoring the distribution of a potentially high-risk clone. Using whole-genome sequencing, we detected multiple introductions of USA300 into eastern England. In terms of epidemiological characteristics, most cases were young and presented with SSTIs to first-opinion services. This is reminiscent of the early disease epidemiology of USA300 in the United States, where this was largely associated with SSTIs in the community before becoming introduced and established through US hospitals [37]. Genomic studies have revealed repeated introductions of USA300 into American cities. The source of the United Kingdom isolates is unknown, but it is likely that international travel has played an important role; one USA300 positive individual was normally resident in the United States. Intercontinental transmission is supported by the similar rates of resistance to oral antibiotics that are commonly used for SSTIs caused by USA300, together with variability in the genome content reflecting the circulating strains in the United States. We identified 2 likely independent acquisitions of ciprofloxacin resistance in addition to those strains that represented the ciprofloxacin-resistant clade that is seen in the United States (Figure 1) [6, 7]. We found no evidence for the introduction of USA300-LV from South America.

USA300 is known to have spread readily through communities in the United States, but we found limited evidence for such transmission in our study. We identified 1 two-member pair and 1 cluster of genetically closely related USA300. We found epidemiological links between all but 1 individual within these groups. Household transmission within pair A and cluster C is supported by findings of previous US studies, in which a median of 3 SNPs (range, 0–772 SNPs), 6 SNPs (range, 0–199 SNPs), and 5 SNPs (range, 0–102 SNPs) were identified per household in New York, Los Angeles, and Chicago, respectively [6, 7, 38].

Our prospective, systematic surveillance study found a prevalence rate of USA300 of 1.6%, which is >3 times higher than the prevalence of MRSA isolates positive for *mecC,* a *mecA* homolog associated with livestock [39]. Serial systematic prevalence studies for USA300 are lacking across Europe, but single hospital studies from Switzerland have shown multiple importations and increasing rates [28], with 1 study showing an increase in isolation rates from 0% to 9.2% between 2002 and 2012 [12]. Like continental European studies, United Kingdom studies are sparse and strongly limited by surveillance methods [10]. The potential for underascertainment within referral laboratory–based infection surveillance studies is apparent when comparing our report to another United Kingdom report; molecular testing in a reference laboratory study identified 40 likely USA300 isolates (CC8 SCC*mec* IVa, *spa* t008, *agr* group 1, PVL positive) from 300 CA-MRSA across England and Wales over a 2-year period (2004–2005) [40], yet we have identified 60% of this total in 1 rather than 2 years and from a single region of England.

The reasons underlying the rapid and widespread dissemination of USA300 in the United States remain unknown, despite hypothesized roles for genetic elements such as ACME and, more recently, the *copB* locus in raising fitness [8, 32, 33]. Phylogenetic analysis has indicated a process of repeated introduction throughout the Americas prior to the epidemic [6, 7]. Our data builds upon other European studies that suggest a similar pattern in Europe, but without systematic surveillance it is difficult to define the trajectory of USA300 in the United Kingdom. In contrast to when USA300 rapidly disseminated in the United States >10 years ago, it is now feasible to implement comprehensive, genomic surveillance strategies to monitor lineage distribution and guide intervention. Had this been possible during the initial stages of the US epidemic, alternative strategies such as an aggressive targeted search-and-destroy policy [41], may have been implemented. Currently, in Europe the majority of countries adopt a reactive rather than active management strategy.

The current methods for surveillance of invasive staphylococcal infections in the United Kingdom do not allow for the monitoring of USA300. Antimicrobial susceptibility profiles have been used as a surrogate marker of community lineages on the basis of greater susceptibility overall, compared with that for previous hospital-adapted lineages. This is prone to increasing inaccuracy over time, since lineages may accumulate drug resistance. For example, in the past, ciprofloxacin susceptibility was used as a marker of United Kingdom community lineages [42], but a substantial amount of resistance to this drug is seen within USA300 (Figure 2). The molecular identification of USA300 remains challenging since putative gene markers such as the ACME element may be lost, and the 4 CAP5 mutations recently described are also seen in the USA300 progenitor, USA500 [27]. The use of whole-genome sequencing for the genetic characterization of USA300 overcomes these barriers. The national Staphylococcus Reference Unit at Public Health England provides a reference service for microbiological characterization of invasive *S. aureus* isolates, which includes *spa* typing, toxin gene detection and whole-genome sequencing. However, isolate submission is currently voluntary, highly selective, and in practice tends to only be used to test isolates associated with life-threatening infection [43]. Review of the local laboratory records showed that only 3 of 56 USA300 isolates were documented as having been submitted to the national reference laboratory and subsequently identified as consistent with potential USA300 strains.

We acknowledge a number of limitations in our study. Without undertaking prevalence studies of all residents within a defined geographical area, studies are biased toward the healthcare-seeking population. We tried to reduce this by including all disease and carriage isolates over a yearlong period. Second, there is likely to be United Kingdom–wide geographic variation in USA300 distribution. Third, ethical constraints mean that epidemiological links between patients have been deduced using electronic medical records, which do not capture all epidemiological links. Despite these limitations, our study has identified the covert presence of USA300 in eastern England. More broadly, it shows that systematic whole-genome sequencing within a sentinel center could function as an effective surveillance mechanism to monitor MRSA lineages. If undertaken systematically, whole-genome sequencing–based sentinel surveillance within a coordinated network could be used to provide an early warning of strain emergence and dissemination; such epidemic intelligence would allow appropriate targeting of resources toward interventions to limit further spread.

## Supplementary Data

Supplementary materials are available at http://jid.oxfordjournals.org. Consisting of data provided by the author to benefit the reader, the posted materials are not copyedited and are the sole responsibility of the author, so questions or comments should be addressed to the author.

Supplementary Data
