# Supplementary material for: Systematic Surveillance Detects Multiple Silent Introductions and Household Transmission of Methicillin-Resistant Staphylococcus aureus USA300 in the East of England
Source: J Infect Dis. 2016 Apr 27;214(3):447–53. doi: 10.1093/infdis/jiw166 (PMC4936647; doi:10.1093/infdis/jiw166)
Supplement: Supplementary Data [file supp_214_3_447__index.html]

Systematic Surveillance Detects Multiple Silent Introductions and Household Transmission of Methicillin-Resistant Staphylococcus aureus USA300 in the East of England — Systematic Surveillance Detects Multiple Silent Introductions and Household Transmission of Methicillin-Resistant Staphylococcus aureus USA300 in the East of England — Supplementary Data 

# Systematic Surveillance Detects Multiple Silent Introductions and Household Transmission of Methicillin-Resistant *Staphylococcus aureus* USA300 in the East of England

## Supplementary Data

Supplementary Data

- Supplementary Data - Pdf file
