# Supplementary material for: Systematic Surveillance Detects Multiple Silent Introductions and Household Transmission of Methicillin-Resistant Staphylococcus aureus USA300 in the East of England
Source: J Infect Dis. 2016 Apr 27;214(3):447–53. doi: 10.1093/infdis/jiw166 (PMC4936647; doi:10.1093/infdis/jiw166)
Supplement: Supplementary Data [file supp_jiw166_jiw166supp.pdf]

Supplementary files:

Table S1: Accession numbers and temporal data for USA300 isolates. *Day 1: The first day of this study.

Figure S1: Map of East of England showing the residences of individuals from which USA300 was isolated, using postcode area. Yellow: one case; orange: two cases; red: three cases. H: hospital location.

Figure S2: Hospital admission and sampling data for individuals with closely related isolates for up to two years prior to study. Each block represents time periods in contact with healthcare. Blue: Admission to specific hospital ward. Green: Visit to specific general practice. Grey: Wards or general practices visited by one individual only. Closed black circles: time of positive MRSA sample collection.

S1: Accession numbers and temporal data for isolates.

*Day 1: The first day of this study.

Study ID	Accession Number	Day Collected*
1	ERR355935	33
2	ERR737219	290
2	ERR715303	295
3	ERR736964	155
3	ERR714975	155
4	ERR701945	132
4	ERR730959	133
5	ERR737332	327
6	ERR213001	83
7	ERR715353	253
7	ERR737176	253
7	ERR715295	281
8	ERR714843	231
8	ERR702215	280
9	ERR212793	10
9	ERR702170	238
9	ERR702210	238
9	ERR702208	280
10	ERR736945	115

10	ERR730953	130
10	ERR774759	167
11	ERR737468	335
12	ERR737164	239
13	ERR736985	134
13	ERR702055	134
13	ERR715270	138
14	ERR737324	321
14	ERR715384	334
15	ERR737105	274
16	ERR737359	135
17	ERR715072	196
18	ERR715053	183
18	ERR737021	184
18	ERR736975	186
18	ERR736978	187
18	ERR715314	189
19	ERR737537	298
20	ERR715099	352
21	ERR714960	152
22	ERR212784	8
22	ERR742877	11
22	ERR737644	12

22	ERR715403	12
22	ERR715191	27
22	ERR715264	27
22	ERR715260	88
22	ERR715227	95
22	ERR715193	95
22	ERR715192	95
22	ERR715262	111
22	ERR742912	155
22	ERR715341	232
22	ERR702200	232
22	ERR737503	344
23	ERR702050	134
24	ERR701919	124

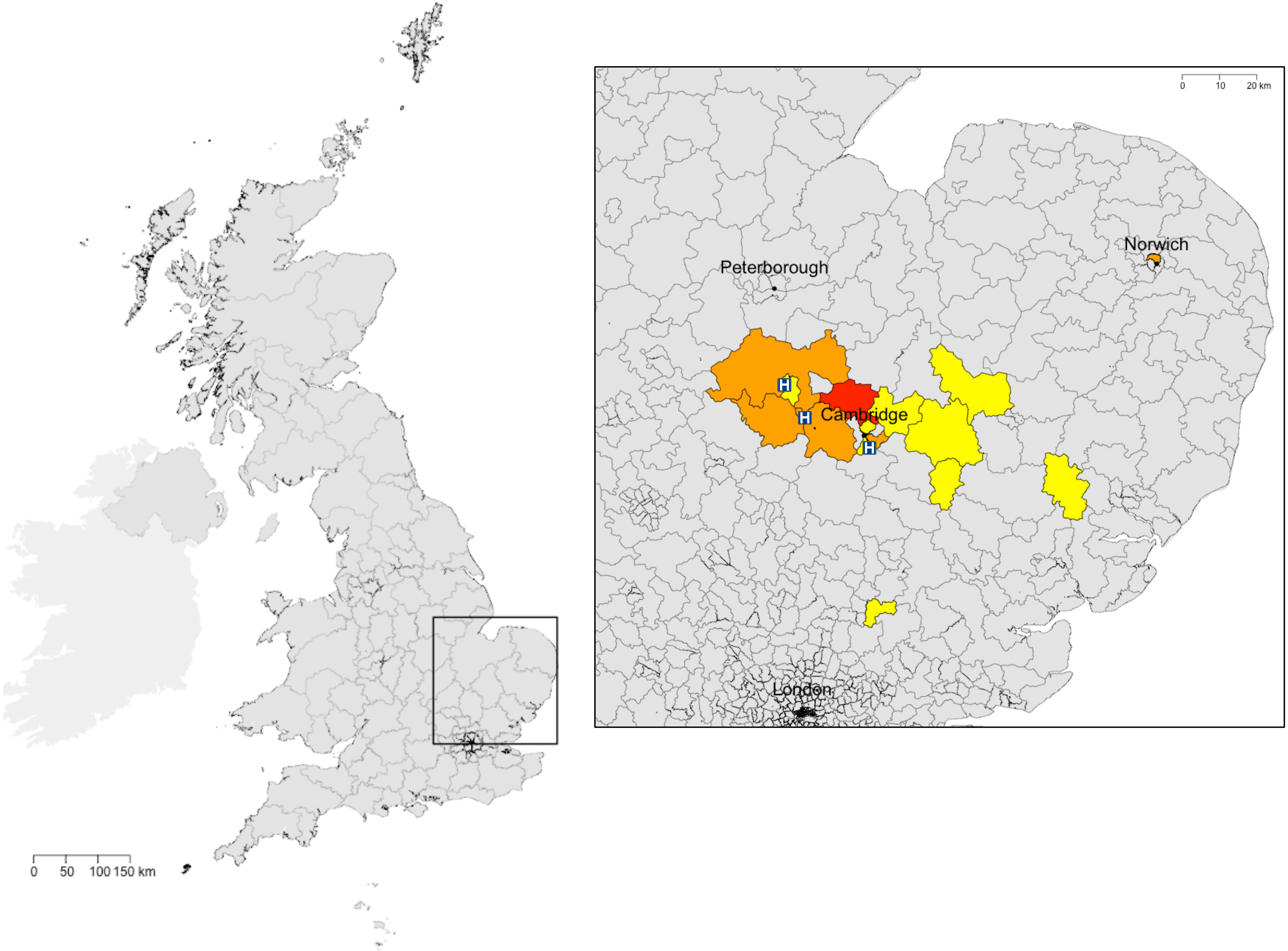
0
50
100 150 km
0
10
20 km
Norwich
Peterborough
Cambridge
London
H

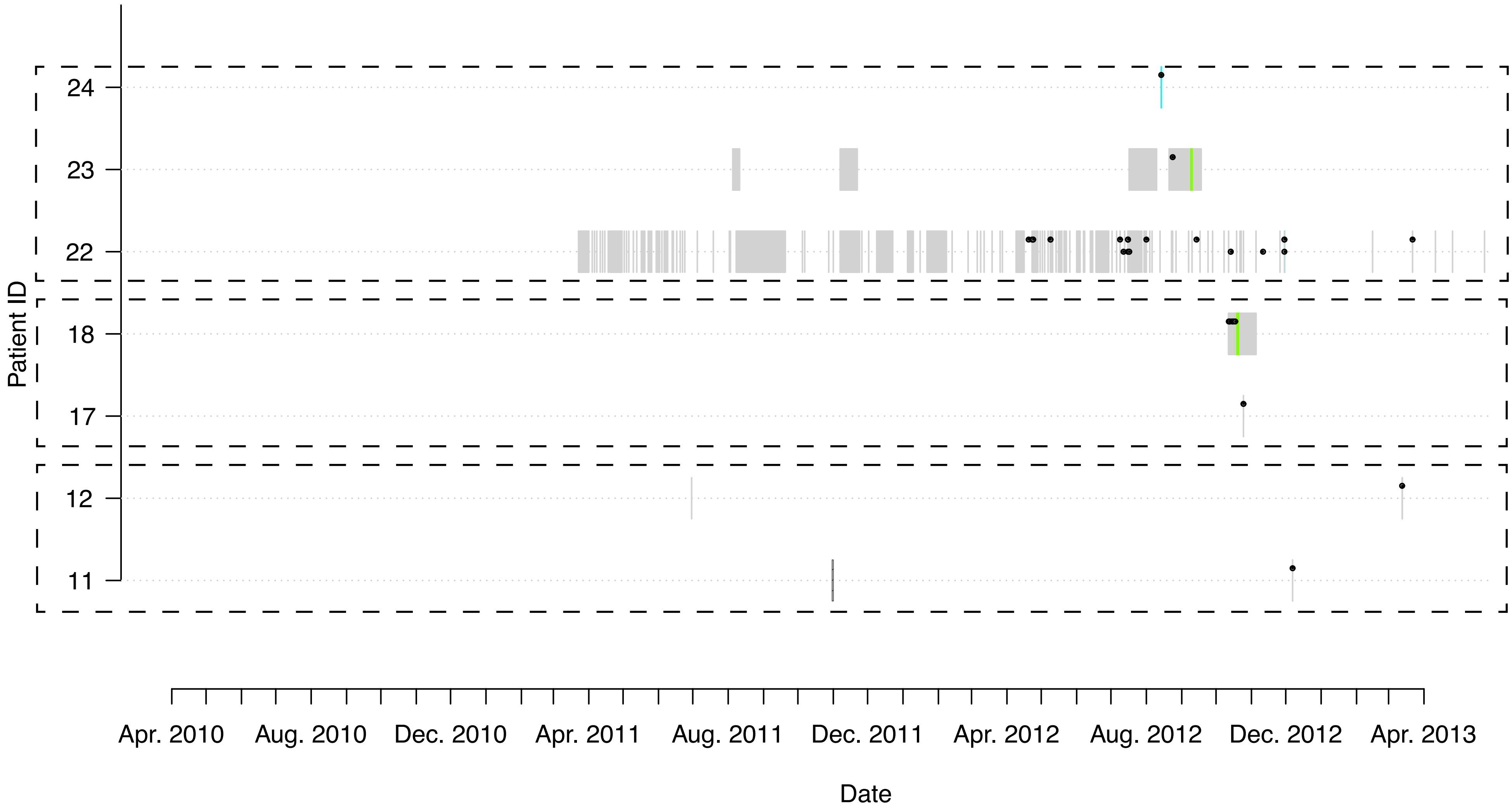
Patient ID
24
23
22
18
17
12
11
Apr. 2010
Aug. 2010
Dec. 2010
Apr. 2011
Aug. 2011
Dec. 2011
Apr. 2012
Aug. 2012
Dec. 2012
Apr. 2013
Date
